# Surgical Management of a Proximal Tibial Epiphyseal Fracture in a Pediatric Patient with Osteogenesis Imperfecta

**DOI:** 10.7759/cureus.84131

**Published:** 2025-05-14

**Authors:** José Juan Villaseñor-Valdés, David Muñoz-Nieto, Edgar Iván García-Estrada, Emmanuel Ramírez-Yañez, Fernando Pérez-Velázquez

**Affiliations:** 1 Orthopedic Surgery, Hospital General de Zona IMSS 7, Monclova, MEX; 2 Orthopedic Surgery, Hospital de Especialidades IMSS Bienestar Dr. Carlos Canseco, Tampico, MEX

**Keywords:** fracture osteosynthesis, open reduction internal fixation, orthopedic management, osteogenesis imperfecta type ii, preoperative planning in io

## Abstract

Osteogenesis imperfecta (OI) is a heritable connective tissue disorder characterized by defective type I collagen synthesis, leading to reduced bone strength and increased susceptibility to fractures, often with minimal trauma. Fractures involving the tibial tuberosity are rare and typically occur in adolescents during periods of rapid growth, usually following high-demand activities. We report a case of a 14-year-old male with type I OI who sustained a displaced tibial tuberosity avulsion fracture with epiphyseal and intra-articular extension (Salter-Harris type III, Ogden type IIIA) following low-energy trauma. Surgical management was undertaken via open reduction and internal fixation using partially threaded lag screws through a medial parapatellar approach, with precautions tailored to the patient’s underlying bone fragility. Postoperative recovery was favorable, and the patient remained under multidisciplinary follow-up. This case emphasizes the importance of individualized surgical planning and technique in patients with OI to ensure stable fixation and minimize complications associated with bone fragility.

## Introduction

Osteogenesis imperfecta (OI) is a rare inherited connective tissue disorder with an estimated incidence ranging from 1 in 10,000 to 20,000 live births [1,2]. Most cases are caused by autosomal dominant mutations in the COL1A1 or COL1A2 genes, encoding type I collagen, a critical structural component of bone matrix [2,3]. The clinical phenotype is heterogeneous and may include recurrent low-energy fractures, bone deformities, short stature, dentinogenesis imperfecta, blue sclerae, and hearing loss [1,4].

From an orthopedic perspective, managing OI remains challenging due to the inherently poor bone quality, narrow medullary canals, and existing deformities, all of which compromise implant fixation and increase surgical risk [5]. Intramedullary rodding, particularly with telescopic devices such as the Fassier-Duval system, has become a standard surgical option in children, aiming to reduce fracture rates and improve functional alignment [6,7]. However, individualized planning is critical, as altered biomechanics and delayed union are common [8,9]. This study describes the management of a proximal tibial epiphyseal fracture (Salter-Harris/Ogden type III) in an adolescent with type I OI. This case illustrates how conventional trauma protocols must be adapted to address the unique anatomical and biomechanical challenges in this population.

## Case presentation

**Figure 1:**
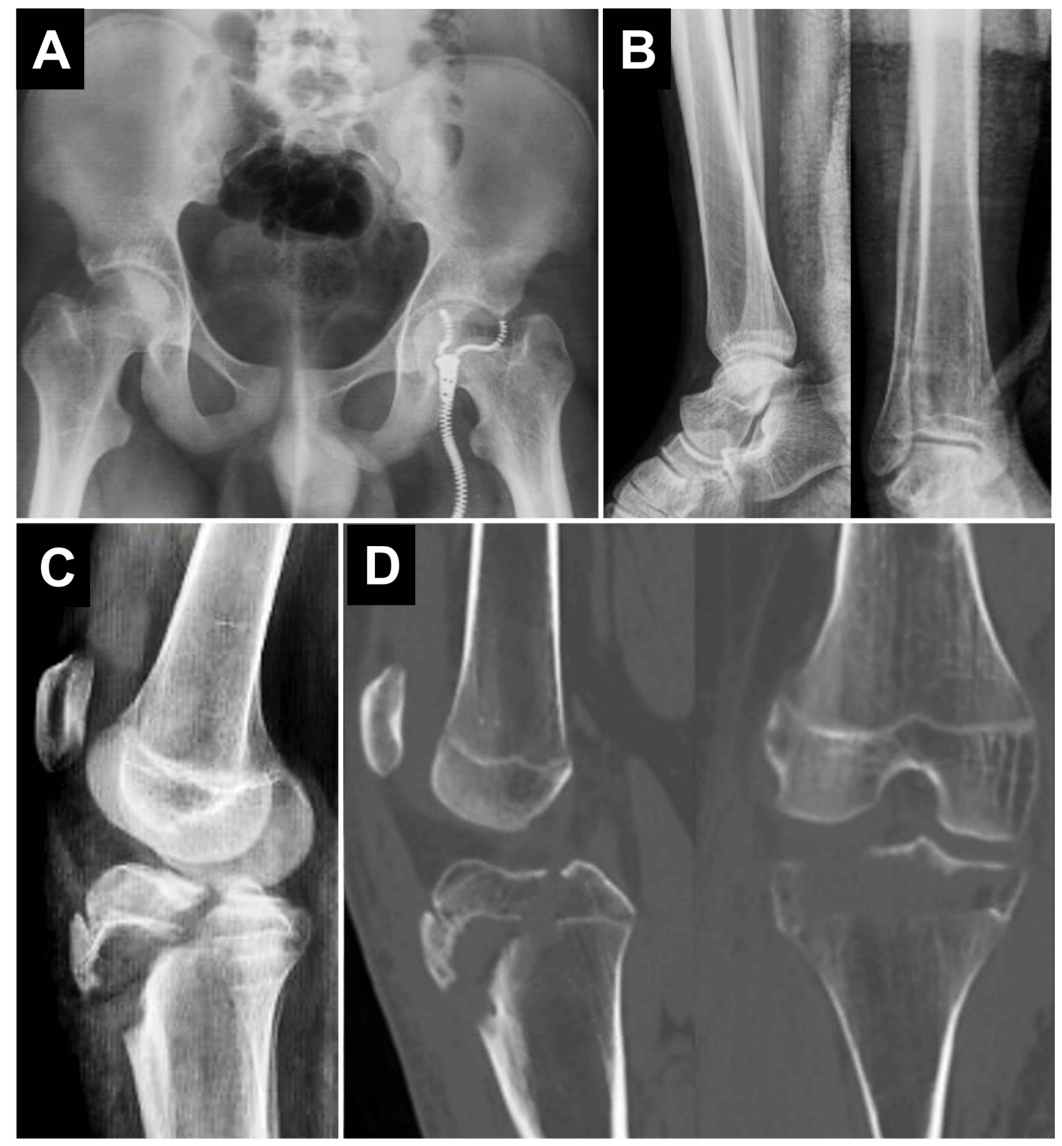
Radiographic overview: historical and preoperative imaging. (A) Anteroposterior pelvic radiograph obtained during previous orthopedic follow-up, demonstrating generalized osteopenia and thin cortical bone, consistent with osteogenesis imperfecta. (B) Prior bilateral ankle radiographs showing metaphyseal modeling and mild deformity. (C) Lateral radiograph of the right knee at presentation, revealing an avulsion fracture of the tibial tuberosity. (D) Coronal and sagittal computed tomography images confirming a Salter-Harris type III (Ogden IIIA) fracture involving the proximal tibial epiphysis with posterior extension into the articular surface, without evidence of comminution or joint incongruity.

A 14-year-old male with a confirmed diagnosis of osteogenesis imperfecta (type I), established during infancy, had a documented medical history of recurrent low-energy fractures since early childhood. He had sustained multiple fractures involving the bilateral tibiae, fibulae, radii, ulnae, and several metacarpal and phalangeal bones. His comorbidities included congenital left-sided sensorineural hearing loss, right lower limb shortening, and obesity. At the time of presentation, the patient was not receiving pharmacologic treatment for OI. Prior imaging studies revealed generalized osteopenia, gracile long bones, and metaphyseal modeling abnormalities (Figures 1A, 1B). He presented to the emergency department after sustaining low-energy trauma to the right lower extremity following contact with another individual and a subsequent fall from standing height. He reported acute, localized pain in the right knee and proximal leg, accompanied by complete functional impairment of the limb. On physical examination, the patient was alert, hemodynamically stable, and in visible discomfort. Inspection revealed localized swelling, mild joint effusion, and ecchymosis over the anterior aspect of the proximal tibia. Palpation elicited marked tenderness over the tibial tuberosity and proximal metaphysis. The range of motion of the knee was severely limited due to pain, particularly during flexion beyond 30°. No joint instability or gross deformity was noted. Neurovascular examination demonstrated intact distal pulses, brisk capillary refill, and preserved motor and sensory function throughout the right lower extremity. Anteroposterior and lateral radiographs of the right knee demonstrated an avulsion fracture of the tibial tuberosity with approximately 11 mm of superior displacement of the osseous fragment. The fracture line extended through the proximal tibial epiphysis without comminution. Computed tomography provided further characterization, confirming a Salter-Harris type III (Ogden type IIIA) fracture with posterior propagation into the articular surface and intercondylar region. No intra-articular fragments, step-off, or joint incongruity were identified (Figures 1C, 1D).

Surgical management was performed the following day under general anesthesia. A medial parapatellar approach was selected, with meticulous handling of soft tissues due to the intrinsic fragility associated with OI. The avulsed tibial tuberosity fragment was identified, mobilized, and anatomically reduced under direct visualization. Internal fixation was performed using a 4.5 × 32 mm and a 6.5 × 16 mm partially threaded lag screw, both inserted in a bicortical manner to achieve interfragmentary compression and ensure rotational stability. Although one screw partially traversed the fracture line, this was deemed necessary to optimize compression in osteoporotic bone and minimize the risk of postoperative fragment displacement. The knee was maintained in 15°-20° of flexion during screw placement to reduce tension on the patellar tendon and minimize apophyseal traction. Special care was taken to avoid stress risers and thermal or mechanical damage to the osteoporotic bone. Intraoperative fluoroscopy confirmed satisfactory reduction, restoration of articular alignment, and stable implant positioning without propagation or implant-related complications. Postoperative management included immobilization in a long leg circular cast with the knee positioned in 20°-30° of flexion to protect the surgical repair and offload the extensor mechanism. The patient was instructed to maintain strict non-weight-bearing status, with the limb elevated, and was closely monitored for signs of compartment syndrome or neurovascular compromise.

**Figure 2:**
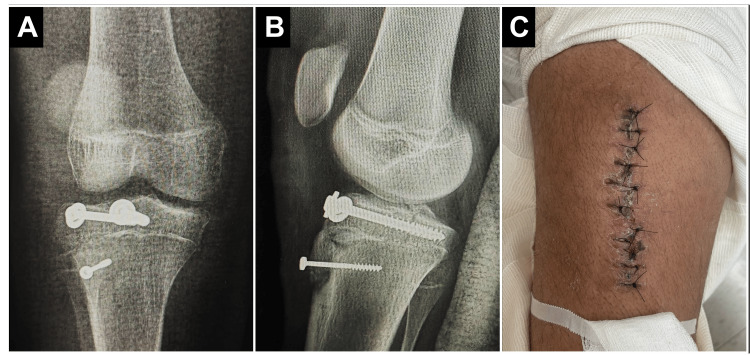
Postoperative outcomes following open reduction and internal fixation. (A) Anteroposterior and (B) lateral radiographs of the right knee showing anatomical reduction and stable fixation of the tibial tuberosity using partially threaded lag screws in bicortical configuration. (C) Clinical image of the right knee demonstrating satisfactory healing of the surgical wound with no signs of infection or dehiscence at early postoperative follow-up.

At the four-week follow-up, the patient remained non-weight-bearing as instructed but was able to ambulate short distances using crutches with splint support. No neurovascular deficits or wound complications were observed. A functional thigh-to-foot splint was maintained, and follow-up radiographs were scheduled for reassessment in two weeks. Cast removal was deferred pending further radiologic assessment of bone healing. Given the underlying diagnosis of osteogenesis imperfecta and the patient’s history of multiple fragility fractures, long-term orthopedic surveillance was planned. Additionally, the patient was referred to pediatric endocrinology for comprehensive metabolic evaluation and to assess the potential indication for bisphosphonate therapy aimed at improving bone mineral density and reducing future fracture risk (Figures 2A-2C).

## Discussion

Fractures involving the tibial tuberosity are uncommon in the pediatric population, accounting for approximately 3% of all proximal tibial fractures [10]. These injuries typically occur in adolescent males between 13 and 16 years of age and are classically associated with eccentric quadriceps contraction during activities involving sudden acceleration or deceleration, such as jumping or sprinting [11]. In the present case, however, the injury resulted from a low-energy direct contusion - an atypical mechanism that highlights the underlying skeletal fragility characteristic of OI. This deviation from typical trauma patterns underscores the biomechanical vulnerability conferred by defective type I collagen, which compromises the tensile strength and load-bearing capacity of cortical bone.

Radiological assessment revealed a displaced avulsion fracture of the tibial tuberosity extending into the articular surface, classified as a Salter-Harris type III and Ogden type IIIA lesion. The vertical extension of the fracture through the physis and epiphysis into the tibial plateau warranted prompt surgical intervention to preserve joint congruency and prevent long-term functional sequelae [12,13]. While conservative management may be appropriate in non-displaced or minimally displaced extra-articular injuries, displaced intra-articular fractures in skeletally immature patients-particularly those with OI-require anatomic reduction and internal fixation to restore joint surface integrity and minimize the risk of physeal damage [12-14]. Open reduction and internal fixation (ORIF) is the preferred approach for displaced Salter-Harris type III fractures with articular involvement, as supported by Hajdu et al. [12], the AO pediatric surgery guidelines [14], and the surgical consensus outlined by Esposito and Plotkin for patients with OI [15]. Closed reduction techniques may be insufficient to restore joint congruity and are associated with an increased risk of physeal injury and growth disturbance in such cases.

In our patient, ORIF was performed via a medial parapatellar approach, with meticulous soft tissue handling to accommodate the inherent fragility associated with OI. Fixation was achieved using partially threaded bicortical lag screws, and anatomical reduction with stable hardware positioning was confirmed intraoperatively under fluoroscopic guidance. Postoperative management included cast immobilization, delayed weight-bearing, and close monitoring for complications such as displacement, growth disturbance, or delayed union. Hardware removal is typically considered within 3-4 months, depending on radiographic evidence of healing and clinical progress [14]. In patients with OI, orthopedic management remains challenging due to impaired bone mineral density, cortical thinning, and altered trabecular structure secondary to collagen synthesis defects. These alterations not only predispose patients to atypical fracture patterns but also increase the risk of surgical complications, including fixation failure, malalignment, delayed union, and growth arrest. Furthermore, bone healing in OI is often unpredictable, requiring extended follow-up and the collaboration of multidisciplinary teams involving orthopedic surgeons, endocrinologists, and rehabilitation specialists [15].

Tibial tuberosity avulsion fractures with epiphyseal extension are rarely described in patients with osteogenesis imperfecta (OI), where bone fragility and atypical fracture morphology complicate management. In the present case, achieving stable fixation required modifying standard techniques to ensure joint congruency and reduce hardware failure risk. The surgical strategy was guided by intraoperative judgment and adapted to the unique structural limitations of OI bone.

## Conclusions

This study illustrates that favorable outcomes can be achieved in patients with OI through tailored surgical planning, precise reduction, and stable fixation-even in the setting of rare physeal fractures. It reinforces the importance of adapting conventional orthopedic approaches to address the unique biomechanical limitations of OI and highlights the value of individualized strategies to reduce complication risk. Ongoing documentation of such cases is essential to refine surgical protocols and improve care for this high-risk population.
